# Major limb replantation: Current status and perspectives

**DOI:** 10.1016/j.jpra.2026.02.005

**Published:** 2026-02-08

**Authors:** Youssef Jaafar, Jack Obeida, Yanis Berkane, Loïc Van Dieren, Elio Nader, Javier Gonzalez, Ali Mojallal, Curtis L. Cetrulo, Alexandre G. Lellouch, Haïzam Oubari

**Affiliations:** a Faculty of Medicine and Medical Sciences, University of Balamand, Youssef Surock Street, Beirut 1100 2807, Lebanon; b Department of Plastic, Reconstructive and Aesthetic Surgery, CHU de Limoges, 2 Avenue Martin Luther King, Limoges 870432, France; c Center for Engineering in Medicine and Surgery, Massachusetts General Hospital, Harvard Medical School, 55 Fruit Street, Boston, MA 02114, USA; d Shriners Children’s Boston, 51 Blossom Street, Boston, MA 02114, USA; e Plastic Surgery Research Laboratory, Department of Plastic and Reconstructive Surgery, Massachusetts General Hospital, Harvard Medical School, 55 Fruit Street, Boston, MA 02114, USA; f Department of Plastic, Reconstructive and Aesthetic Surgery, CHU de Rennes, University of Rennes, 16 Boulebard de Bulgarie, Rennes 35203, France; g Faculty of Medicine and Health Sciences, University of Antwerp, Campus Die Eiken, Wilrijk 2610, Belgium; h Poznan University of Medical Sciences, Faculty of Medicine, Ul Fredry 10, Poznan 61701, Poland; i Division of Plastic Surgery Research, Vascularized Composite Allotransplantation Program Development, Cedars-Sinai Hospital, 8635 W. 3rd St, Los Angeles, CA 90048, USA; j Department of Plastic, Reconstructive and Aesthetic Surgery, Hopital de la Croix Rousse - Hospices Civils de Lyon, 103 Grande Rue, Lyon 69004, France

**Keywords:** Major limb replantation, Ischemia-reperfusion injury, Microsurgery, Ex vivo perfusion, Limb preservation, Traumatic amputation

## Abstract

**Background:**

Major limb replantation is a demanding microsurgical procedure aiming to restore form and function after traumatic amputation. Although survival has improved since the 1960s, ischemia, infection, and complex reconstruction continue to limit outcomes. Emerging preservation and reconstructive strategies may help expand indications and improve results.

**Methods:**

For this scoping review, MEDLINE, Embase, and the Cochrane Library were searched from inception through December 2024 using predefined MeSH terms related to major limb replantation. Adult case series and reports were included; pediatric cases and long-term follow-up studies were excluded. Screening and data extraction were conducted independently by three reviewers, with consensus resolution of discrepancies.

**Results:**

Ninety-nine publications reported outcomes in 1107 patients with traumatic amputations. The mean age was 31.6 years (range 17–63). Upper-limb replantation represented 88% of cases, most commonly at the forearm and elbow. Mean cold ischemia time was 10 ± 8 h and mean warm ischemia time 4.4 ± 2.3 h. Static cold storage was predominant (72%), while ex vivo dynamic perfusion and ectopic replantation were selectively applied. Overall replantation success was 68%, highest after sharp amputations (85%) and lower following crush (45%) and avulsion (55%) injuries. Vascular complications, infection, and systemic inflammatory response accounted for most failures.

**Conclusions:**

Major limb replantation remains constrained by ischemia-reperfusion injury and surgical complexity. New preservation techniques, adjunctive microsurgery, and optimized perioperative care show potential to improve viability and function. Continued multidisciplinary advancement is essential to strengthen outcomes and accessibility in both civilian and military trauma care.

## Introduction

Major limb replantation is a technically demanding microsurgical procedure that aims to restore both form and function following traumatic amputation. Advances in microsurgical techniques have significantly improved limb survival and functional recovery.^1^ However, despite decades of progress, replantation of major limbs continues to present clinical and logistical challenges,^2^ with outcomes highly dependent on a variety of factors, including ischemia duration, mechanism of injury, degree of contamination, and the presence of associated systemic trauma.^3^^,^^4^ Among these variables, ischemia time remains one of the most critical determinants of successful replantation.^5^ Prolonged warm or cold ischemia can cause irreversible tissue damage, jeopardizing graft viability and leading to poor outcomes. Likewise, injuries involving contamination, especially in high-energy mechanisms such as crush or avulsion injuries, further increase the risk of infection, vascular thrombosis, and soft-tissue necrosis.^6^ These risks often necessitate complex adjunctive procedures, including free tissue transfer, bone shortening, and nerve grafting, to optimize reconstruction and improve long-term function. Consequently, only large and well-equipped hospitals are best positioned to perform these procedures, which sometimes require long-distance repatriations. This contributes to explaining why replantation is reported in only about one out of five cases of limb amputation.^7^ Recent innovations in limb preservation, particularly ex vivo perfusion technologies, offer promising solutions for extending ischemia tolerance and enhancing tissue viability prior to replantation. By delivering oxygenated perfusates and antimicrobial agents, these systems aim to reduce ischemia-reperfusion injury and facilitate delayed replantation in complex or otherwise non-salvageable cases.^8^ Additionally, adjunctive microsurgical techniques, such as the use of free flaps for soft-tissue reconstruction, have significantly broadened the indications for replantation and improved outcomes in previously contraindicated scenarios. This scoping review provides a comprehensive synthesis of the current evidence surrounding major limb replantation. It highlights key determinants of surgical success, including ischemia time, preservation strategies, mechanisms of injury, and complication profiles. Furthermore, we discuss the evolving role of emerging techniques and future directions that could redefine limb salvage in both civilian and military trauma settings.

## Methods

This scoping review was conducted in accordance with the Preferred Reporting Items for Systematic Reviews and Meta-Analyses (PRISMA) guidelines. A systematic literature search was performed using the electronic databases MEDLINE (PubMed), Embase, and the Cochrane Library, covering all records from inception through December 2024. The search strategy included the following MeSH terms: ((major limb) OR (leg) OR (arm) OR (hand) OR (foot) OR (lower limb) OR (upper limb) OR (extremity) OR (forearm)) AND (replantation). To ensure thorough coverage, reference lists of relevant articles and reviews were manually screened for additional eligible studies. The search was restricted to articles published in English, and duplicates as well as non-English studies were excluded during the initial screening of titles and abstracts. All references retrieved from the database searches were exported to the Rayyan screening tool (Rayyan Systems Inc., Cambridge, MA, USA), which was used to manage reference lists and identify duplicate records.

All reports and case series of major limb replantation in adult patients (≥17 years) having sustained a traumatic amputation were eligible for inclusion. This review only considered amputations at the wrist or ankle level, or more proximal, representing major limb amputations; digital or toe amputations were excluded, as only cases involving substantial muscle mass were included in the analysis. The review focused on studies reporting key aspects of acute management and early postoperative outcomes, excluding long-term outcomes.

The selection process involved three independent reviewers (JO, YJ, and HO) who screened the titles and abstracts of identified articles to determine their eligibility based on the predefined inclusion and exclusion criteria using the Rayyan platform. Disagreements between reviewers were resolved through discussion, and full-text articles of potentially eligible studies were obtained for detailed review.

Data from the included studies were extracted using a standardized Excel (Microsoft Corporation, Redmond, WA, USA) data extraction form. The collected information included study characteristics (e.g., author, year, country), patients’ demographics, level and mechanism of amputation, adjuvant techniques, and assessed early outcomes. Quality assessments were conducted independently, and any differences were resolved through discussion and, when necessary, with the input of a senior author (AGL, CLC, HO). A narrative synthesis of the included studies was performed, with data organized to highlight the key findings.

## Results

**Figure 1:**
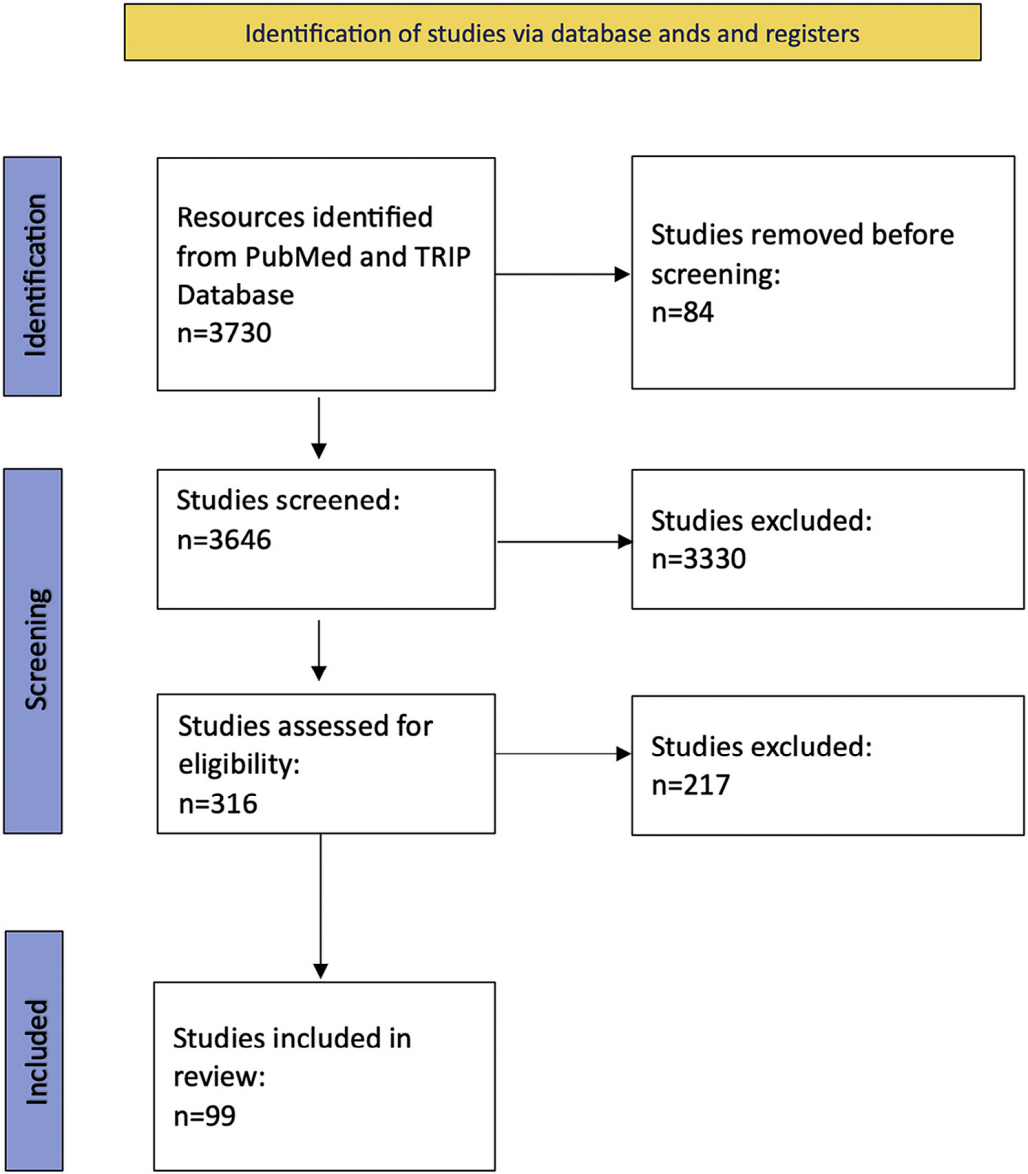
Flowchart.

An initial search of PubMed and TRIP Database sources identified 3730 articles. After removing duplicates and screening titles, abstracts, and full-text reviews for relevant records, 99 were selected and included in this systematic review (Figure 1). All the included studies were case series published between 1984 and 2024. The mean number of participants per study was 10.6 ± 26.6 SD, ranging from 1 to 165.

### Demographics

The study included 1107 participants, of whom 81.9% were male and 18.1% female. Patient ages ranged from 17 to 63 years, with a mean age of 31.6 ± 12.28 years. The majority of cases were reported in Europe (34%) and Asia (31%), while North America contributed a smaller proportion (17%). Upper limb amputations accounted for 88% of cases, most frequently involving the forearm. Lower limb amputations represented 7% of cases, with the most common level of amputation occurring in the lower leg (distal tibia). A small subset of cases were categorized as ‘other’, encompassing multi-level, proximal, or ambiguously reported amputations that could not be reliably assigned to standard anatomic regions (Table 1).

**Table 1:** Age, sex and location of replantation.

	Arm	Forearm	Hand	Leg	Other	Total
Total number of cases	5	950	31	82	39	1107
Mean Age [**Min–Max**] (years)	25.0 [19.0–32.0]	31.70 [19.0–50.0]	29.7 [17.0–51.0]	30.8 [17.0–57.0]	33.0 [17.0–63.0]	31.6 [17.0–63.0]
Male (%)	75.0 (%)	78.0 (%)	77.4 (%)	83.3 (%)	89.3 (%)	81.9 (%)
Female	25.0 (%)	22.0 (%)	22.6 (%)	16.7 (%)	10.7 (%)	18.1 (%)

#### Limb preservation

The average warm ischemia time was 4.4 ± 2.3 h, while the average cold ischemia time was 10 ± 8 h. Three main types of limb preservation were used: ex vivo static preservation, ex vivo dynamic preservation, and transient ectopic replantation.

The most frequently reported method was static cold storage, accounting for the vast majority of cases. In hypothermic ex vivo static preservation, limbs were stored at 4°C in ice water or coolers after being placed in sterile amputation bags or wrapped in saline-soaked gauze. The mean preservation duration with this technique was 8 h, ranging from 120 to 720 min. Although static cold storage was explicity reported in 72% of cases, an additional 23% of reports did not specify the preservation method used.

Only one report by Greaney et al. described the use of an extracorporeal membrane oxygenation (ECMO) circuit to perfuse a traumatically amputated upper extremity in a 19-year-old patient.^9^ After an initial attempt at replantation was aborted due to hemorrhagic complications, the amputated limb was connected to the ECMO circuit and maintained for approximately 72 h, with preserved capillary refill and muscle responsiveness during the support period. Ultimately, replantation was not completed due to progressive muscle demarcation and systemic infection, but the case demonstrated that temporary extracorporeal perfusion can maintain limb viability beyond conventional ischemia limits.

In addition to ex vivo preservation, ectopic implantation and transient catheter replantation are alternatives to orthotopic replantation and have been reported in a small subset of cases. Following, Liaghat et al. reported a case of banking a complete hand at the ankle for 8 days before definitive replantation, which resulted in useful functional recovery at 30-month follow-up.^10^ In lower extremities, Wang et al. described temporary implantation of amputated legs using vessels of the anterolateral thigh flap, allowing delayed replantation after systemic stabilization.^11^ Numerous recipient sites have been reported for temporary replantation, including the thigh,^12^^,^^13^ ankle and foot,^10^ thoracic wall and groin.^14^ Across these reports, survival rates of ectopically banked major limbs were high (>80%), after a mean of 5.7 h (3.5–8 h) of ischemia before replantation, although secondary procedures such as vein grafting, free flap coverage, or staged reconstructions were often required. In most cases, the duration of transient heterotopic replantation was approximately 1 week, but could extend to several months in some cases. Alternatively, adjunctive technical refinements such as temporary catheter perfusion for 10–20 min and artery-last repair sequences further extended the ischemia^15^^,^^16^ tolerance of macroreplantations and facilitated complex skeletal and soft-tissue repairs. Thus, while transient replantation accounted for only a small portion of major limb replantations in our review, it consistently provided a safe bridge to definitive replantation in hemodynamically unstable patients or in cases with severe contamination, and in select circumstances was even employed as a permanent reconstructive strategy.

#### Replantation and adjunct procedures

Patient stabilization was critical for successful replantation. According to the studies, 30% of patients were prepared for immediate replantation upon arrival. However, due to complications such as severe shock, hemorrhage, or multiple traumas, 66% required stabilization prior to replantation. In many cases, pre-existing medical conditions such as diabetes, peripheral vascular disease, and chronic kidney disease further increased the risk of replantation failure.^17^ Intensive therapy was a common component of stabilization to address underlying trauma and manage fluid and blood loss. Blood transfusions, fluid resuscitation, and targeted treatment of associated injuries were among the frequently employed interventions. Replantation was most often contraindicated in patients presenting with multiple organ injuries, hypovolemic shock, or uncontrolled bleeding.^3^^,^^4^

**Figure 2:**
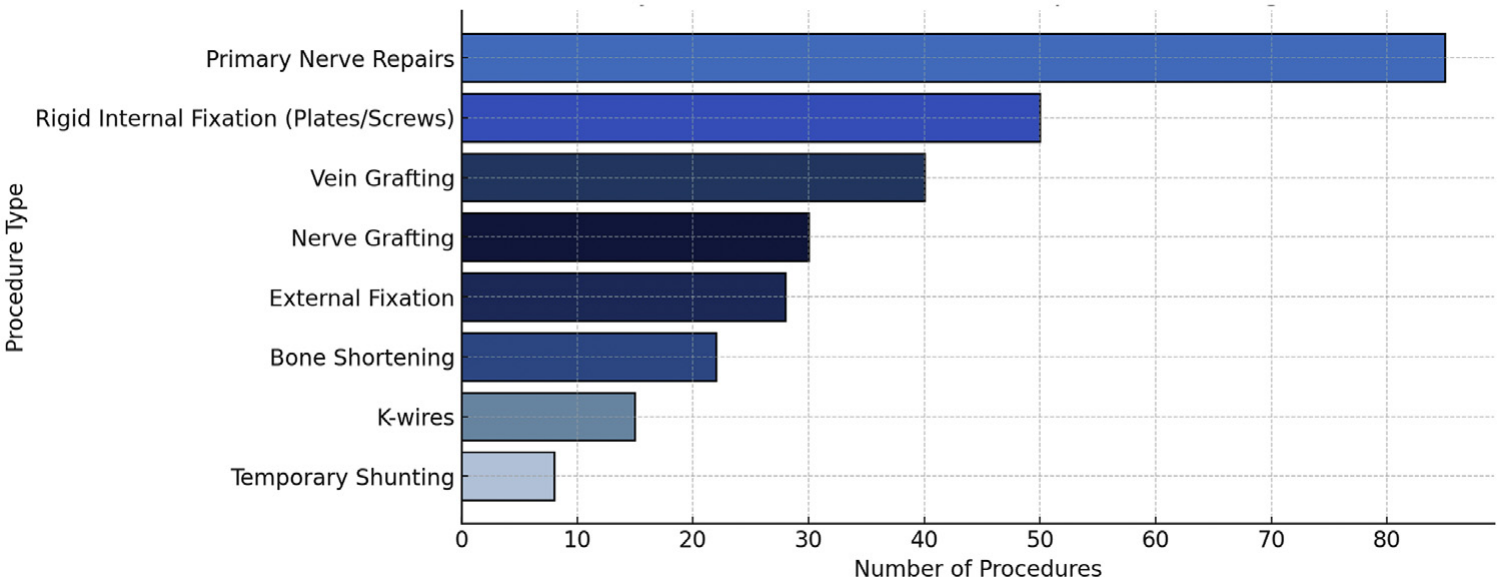
Main procedure in limb replantation.

Furthermore, adjunct procedures were frequently required, representing a substantial proportion of the surgical interventions performed (Figure 2). Osteosynthesis was essential in most replantation cases, providing the foundation for effective bone stabilization. Rigid internal fixation with plates and screws was required in 40% of cases to ensure stability and proper alignment. External fixation was used in 25% of cases, particularly for complex injuries where internal fixation was less suitable due to extensive soft tissue damage or contamination. K-wires, represented 15% of stabilization techniques. Additionally, bone shortening was performed in 20% of cases, with a reduction range of 2 to 5 cm, to facilitate tension-free soft tissue and vascular repairs.

Vascular repair was a key element in replantation procedures, and in most cases consisted of end-to-end arterial and venous orthotopic anastomoses. Vein grafting was used to correct vessel length disparities and guarantee proper vascular connections in 30% of cases. The greater saphenous vein was the most commonly used conduit in the lower limb replantations,^4^ while superficial upper extremity veins such as cephalic and basilic veins were preferred in upper limb replantations.^17^ Temporary shunting methods like femoral-radial shunts to limit ischemia durations and maintain tissue viability during intricate reconstructions were also used in 5% of cases. In 85% of cases, primary nerve repair was complete by direct and end-to-end suture. In 25% of cases, nerve grafting was used to fill in gaps and hasten sensory and motor recovery in patients with segmental nerve defects.

Some authors report the use of free flap transfers as adjunct procedures in cases with extensive soft-tissue loss.^19^, ^20^, ^21^ Free flaps were used primarily to cover neurovascular structures,^19^ achieving immediate soft tissue closure,^21^ and avoiding the need for follow-up procedures like delayed reconstruction or bone grafting by using bone-containing free flaps.^20^ The main flaps used included the latissimus dorsi (LD),^22^ transverse rectus abdominis musculocutaneous (TRAM) ,^23^ and anterolateral thigh (ALT) flaps.^19^ Free flaps were more frequently utilized in lower limb replantations (42%) compared to upper limb cases (15%), particularly in the setting of high-energy trauma causing extensive soft-tissue damage, frequently associated with distal bone exposures. Skin grafts were used in approximately 25.2% of cases, either for primary soft tissue cover or muscular flap coverage, with a notable preference for their use in lower limb injuries.^24^

#### Outcomes

The overall success rate of limb replantation is 68%, with the highest success observed in sharp amputations (85%). In contrast, significantly lower success rates are reported for crush injuries (45%) and avulsion injuries (55%), reflecting the greater complexity and severity of tissue damage in these cases. Systemic complications, infections, and vascular issues remain the predominant causes of failure.

Among systemic complications, systemic inflammatory response syndrome (SIRS) emerges as a notable concern, affecting 20% of patients, particularly in those subjected to prolonged ischemia and extensive soft tissue trauma. These inflammatory responses tended to require more aggressive medical management, prolonged hospital stays, and close hemodynamic monitoring.^5^^,^^25^^,^^26^ Infections are the most frequently reported postoperative complication, occurring in 34% of cases, with superficial infections in 22%, typically managed with antibiotics, while deep infections are observed in 10% and often necessitate surgical debridement. Soft tissue necrosis (15%) is particularly prevalent in patients with extensive soft tissue loss, further impairing wound healing and limb viability.

**Figure 3:**
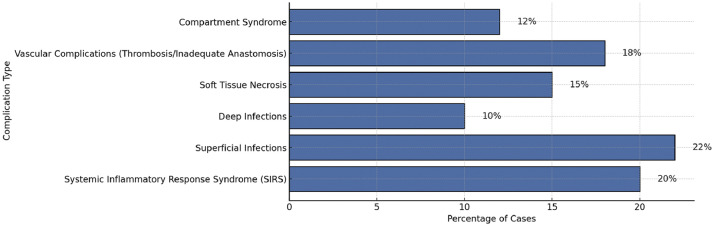
Complications in limb replantation.

**Figure 4:**
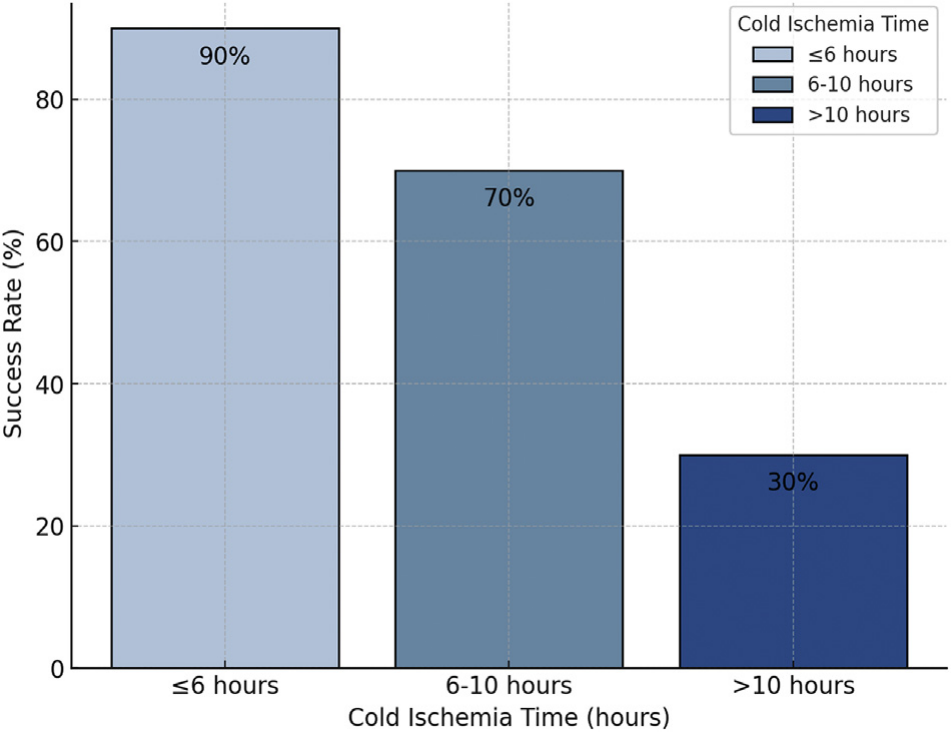
Impact of ischemia time on success rate.

Vascular complications, including thrombosis and anastomosis failure, are reported for 18% of patients, frequently necessitating urgent revision surgeries to restore perfusion and prevent limb loss. These revisions were attempted in 81% of cases and were successful in 67% of the cases.^4^^,^^18^^,^^27^ Compartment syndrome was reported in 12% of cases, requiring timely fasciotomies to alleviate pressure and avert further ischemic damage (Figure 3). Notably, ischemia time emerged as a critical determinant of surgical success, with outcomes deteriorating sharply when warm ischemia exceeded 6 h or cold ischemia extended beyond 10 h (Figure 4).

## Discussion

High-energy mechanisms in both civilian and military settings account for most traumatic amputations and define the challenging context in which replantation is considered.^28^^,^^29^ In our review, the overall replantation success rate was 68%, with the best outcomes observed after sharp amputations (85%) and markedly lower rates in crush and avulsion injuries (45–55%), reflecting their greater soft-tissue and microvascular burden. Major limb replantation remains constrained by prolonged ischemia, complex vascular repairs, and a high risk of infection due to contamination of the amputated segment. These factors compromise graft viability and increase systemic complications, sometimes prompting a decision against replantation. Early failures were primarily related to infection (34%), vascular compromise (18%), soft-tissue necrosis (15%), and systemic inflammatory responses (20% SIRS), underscoring the need for coordinated microsurgical and critical-care management. High-energy trauma further exacerbates these risks through extensive tissue loss, vascular disruption, and prolonged ischemia, often requiring adjunct procedures such as vein grafting, free-flap coverage, or staged reconstructions.^6^^,^^27^^,^^30^ Their success remains time-sensitive and contingent on both limb condition and patient stability. Beyond the surgical challenges, logistical factors are equally decisive: in military settings, delayed evacuation and resource limitations frequently preclude timely replantation,^31^ while in civilian systems, outcomes depend on access to specialized teams and referral networks, with persistent regional disparities.^2^ Collectively, these findings highlight that major limb replantation is not solely a microsurgical procedure but a multidisciplinary endeavor requiring coordinated trauma systems, rapid transfer protocols, and dedicated centers of excellence to achieve optimal outcomes.

Ischemia–reperfusion injury (IRI) remains the dominant biological constraint, with outcomes deteriorating beyond 6 h of warm and 10 h of cold ischemia.^5^ Standard static cold storage continues to predominate, while ectopic banking and transient catheter perfusion offer pragmatic bridges in contaminated or hemodynamically unstable cases.^10^^,^^11^^,^^13^, ^14^, ^15^, ^16^, ^17^ These strategies expand the reconstructive window but demand staged planning (vein grafts, free-flap coverage, nerve repair) and robust ICU support. Emerging ex vivo machine perfusion (MP) is a credible avenue to extend preservation and actively recondition limbs, attenuating IRI, enabling targeted antimicrobials, and even facilitating ex vivo thrombolysis and diagnostics.^32^, ^33^, ^34^, ^35^, ^36^, ^37^, ^38^, ^39^, ^40^, ^41^, ^42^ Early swine,^43^, ^44^, ^45^, ^46^ human-limb,^47^^,^^48^ and VCA data, suggest MP can maintain viability for 24 h+ and potentially several days.^8^^,^^49^ These advances in ex vivo perfusion platforms not only open new perspectives for extending preservation times but also enable active and functional manipulation of tissues prior to replantation.^50^, ^51^^–^^52^ In this context, ex vivo reconditioning and reengineering emerge as promising strategies to enhance tissue viability and improve replantation outcomes. For instance, endothelial stabilization through perfusion-based delivery of targeted agents, such as CD11b inhibitors, has been proposed to mitigate ischemia–reperfusion injury and preserve microvascular integrity.^53^^,^^54^ However, translation will hinge on device simplicity, cost containment, and protocol standardization, as well as prospective human data demonstrating functional benefit and cost-effectiveness. Ex vivo preservation requires sophisticated perfusion systems and interdisciplinary expertise, and their widespread adoption will depend on increased funding, streamlined regulation, and cost-effective implementation. Emerging technologies such as artificial intelligence and machine learning may further optimize surgical planning, patient selection, and outcome prediction.^41^^,^^42^

Logistics remain as decisive as technique: time to care, regional access to microsurgical teams, and referral pathways critically influence candidacy and outcomes.^2^^,^^33^ Regionalized replantation networks with rapid transfer protocols and early involvement of reconstructive microsurgery and critical care teams are pivotal to improving results. There remains a pressing need to develop preservation platforms capable not only of maintaining limb viability but also of ensuring the simultaneous stabilization of both the amputated limb and the patient. However, translating these advances into clinical practice remains constrained by high costs, technical complexity, and variable outcomes. If successfully translated into clinical practice, ex vivo reconditioning technologies such as machine perfusion should be evaluated in trials that prioritize clinically meaningful endpoints, including graft salvage, complication and revision rates, functional recovery, and patient-reported outcome measures, alongside comprehensive health-economic analyses. Major limb replantation continues to offer meaningful salvage for selected patients, but success ultimately depends on the mechanism of injury, ischemia control, infection prevention, and coordinated systems of care. Adjuncts such as ectopic banking are valuable in complex presentations, and machine perfusion represents a promising, yet still maturing, platform to broaden indications and improve outcomes.

By design, this scoping review focused on early outcomes. An important limitation is the absence of a dedicated analysis of long-term functional outcomes following major limb replantation, as these outcomes are multifactorial and time-dependent, reflecting not only the success of the initial surgical procedure but also adjunctive interventions, rehabilitation strategies, and multiple patient- and system-related cofactors.^55^, ^56^, ^57^, ^58^ To validate novel replantation strategies such as anti–ischemia–reperfusion injury and ex vivo preservation, future studies should prioritize standardized functional assessments and patient-reported outcome measures, alongside long-term follow-up, to more comprehensively define success after major limb replantation and better inform patient selection and counseling.

## Conclusion

Major limb replantation remains an invaluable reconstructive option, offering the potential for functional and psychological restoration following devastating traumatic amputations. While advances in microsurgical technique and perioperative management have substantially improved outcomes, challenges such as prolonged ischemia, infection, and systemic complications continue to hinder success. Emerging strategies, including ex vivo dynamic perfusion, adjunctive free tissue transfer, and improved ischemia-reperfusion modulation, hold promise for expanding the therapeutic window and enhancing limb viability. Future research must focus on integrating these innovations with streamlined patient selection criteria, improved critical care pathways, and cost-effective technologies to make replantation more accessible and successful in both civilian and military settings. A concerted, multidisciplinary effort will be critical to overcoming current limitations and ensuring that patients benefit from the best available reconstructive options.

## Funding

This research received no specific grant from any funding agency in the public, commercial, or not-for-profit sectors.

## Author contributions

YJ and JO contributed equally to the conception, data collection, and drafting of the manuscript. HO and AGL supervised the project. All authors contributed to data interpretation, critical revisions, and approved the final version.

## Ethical approval

Not applicable (literature review).

## Patient consent

Not applicable.

## Declaration of competing interest

The authors declare no conflicts of interest.

## References

[1] Tessler O., Bartow M.J., Tremblay-Champagne M.P. (2017). Long-term health-related quality of life outcomes in digital replantation versus revision amputation. J Reconstr Microsurg.

[2] Stögner V.A., Hauc S.C., Hosseini H. (2025). A nationwide analysis on major upper extremity amputations and replantations. Hand (NY).

[3] Hierner R., Betz A.M., Comtet J.J. (2007). Indications and contraindications in replantation surgery. Eur J Trauma Emerg Surg.

[4] Battiston B., Tos P., Pontini I., Ferrero M. (2002). Primary microsurgical reconstruction of complex upper limb injuries. Injury.

[5] Paradis S., Charles A.L., Meyer A. (2016). Chronology of mitochondrial and cellular events during skeletal muscle ischemia-reperfusion. Am J Physiol - Cell Physiol.

[6] Brown K.V., Murray C.K., Clasper J.C. (2010). Infectious complications of combat-related mangled extremity injuries in the British military. J Trauma.

[7] American Society for Surgery of the Hand (2023). ASSH/ACS national hand truama center. https://www.assh.org/s/hand-trauma-center-network.

[8] Oubari H., Van Dieren L., Berkane Y. (2025). Development of a 24-h preservation protocol of forearm vascularized composite allotransplants in nonhuman primates using subnormothermic machine perfusion. Transplant Direct.

[9] Greaney P.J., Cordisco M., Rodriguez D., Newberger J., Legatt A.D., Garfein E.S. (2010). Use of an extracorporeal membrane oxygenation circuit as a bridge to salvage a major upper-extremity replant in a critically ill patient. J Reconstr Microsurg.

[10] Liaghat O., Shabbooie Z. (2020). Ectopic banking and implantation of an amputated hand. Indian J Orthop.

[11] Wang K.C., Hung K.S., Chang T.Y., Wu P.T., Lee Y.C. (2019). Temporary ectopic implantation of an amputated leg using the distal runoff vessel of the anterolateral thigh flap followed by subsequent prefabricated chimeric replantation. Ann Plast Surg.

[12] Wang J.N., Tong Z.H., Zhang T.H. (2006). Salvage of amputated upper extremities with temporary ectopic implantation followed by replantation at a second stage. J Reconstr Microsurg.

[13] Cavadas P.C., Landin L., Navarro-Monzones A., Soler-Nomdedeu S. (2006). Salvage of impending replant failure by temporary ectopic replantation: a case report. J Hand Surg.

[14] Nazerani S., Vaseghi H., Hesami S., Nazerani T. (2013). Ectopic major transplantation for salvage of upper and lower extremity amputations. Chin J Traumatol Zhonghua Chuang Shang Za Zhi.

[15] Cavadas P.C., Landín L., Ibáñez J. (2009). Temporary catheter perfusion and artery-last sequence of repair in macroreplantations. J Plast Reconstr Aesthetic Surg JPRAS.

[16] Chin K.Y., Hart A.M. (2012). Temporary catheter first perfusion during hand replantation with prolonged warm ischaemia. J Plast Reconstr Aesthetic Surg JPRAS.

[17] Cavadas P.C., Landin L., Ibáñez J. (2007). Ectopic replantation and revascularization of extremities: a series of 10 cases. J Reconstr Microsurg.

[18] Morrison W., O’Brien B., MacLeod A. (1981). Replantation of lower limb amputations: a review of 29 cases. J Trauma.

[19] Wei F.C., Jain V., Celik N., Chen H.C., Chuang D., Lin C. (2004). Selection of recipient vessels in microsurgical free tissue transfer to the lower extremities. Plast Reconstr Surg.

[20] Koshima I., Soeda S. (1995). Free vascularized fibula grafts with skin and muscle for reconstruction of extensive composite bone and soft-tissue defects. Microsurgery.

[21] Chen H.C., Tang Y.B. (2007). Soft-tissue coverage of the lower leg with free flaps. Plast Reconstr Surg.

[22] Yazar S., Lin C.H., Lin Y.T., Wei F.C. (2006). Outcome comparison between free ALT and free latissimus dorsi flaps in reconstruction of massive lower extremity defects. Plast Reconstr Surg.

[23] Khouri R.K., Shaw W.W. (1989). Reconstruction of the lower extremity with microvascular free flaps: a 10-year experience with 304 consecutive cases. Plast Reconstr Surg.

[24] Zhu S., Xu D.C., Li Y.Y., Zhang H., Chen X., Wang Y. (2020). Long-term outcomes of limb replantation after traumatic amputation: a 10-year retrospective study. J Reconstr Microsurg.

[25] Kaltenborn A., Beyer J., Ring A. (2020). Ex vivo limb perfusion for traumatic amputation in military medicine: a feasibility study. Mil Med Res.

[26] He J., Khan U.Z., Qing L., Wu P., Tang J. (2022). Improving the ischemia-reperfusion injury in vascularized composite allotransplantation: clinical experience and experimental implications. Front Immunol.

[27] Godina M. (1986). Early microsurgical reconstruction of complex trauma of the extremities. Plast Reconstr Surg.

[28] Ziegler-Graham K., MacKenzie E.J., Ephraim P.L., Travison T.G., Brookmeyer R. (2008). Estimating the prevalence of limb loss in the United States: 2005 to 2050. Arch Phys Med Rehabil.

[29] Stansbury L.G., Lalliss S.J., Branstetter J.G., Bagg M.R., Holcomb J.B. (2008). Amputations in U.S. military personnel in the current conflicts in Afghanistan and Iraq. J Orthop Trauma.

[30] Henn R.F., Kloen P., Helfet D.L. (2004). Extremity soft tissue coverage: current practice and future directions. J Am Acad Orthop Surg.

[31] Kaltenborn A., Krezdorn N., Hoffmann S. (2020). Ex vivo limb perfusion for traumatic amputation in military medicine. Mil Med Res.

[32] Oubari H., Berkane Y., Lellouch A. (2025). Response to: “machine perfusion organ preservation: highlights from the american transplant congress 2024. Artif Organs.

[33] Berkane Y., Filz von Reiterdank I., Tawa P. (2024). VCA supercooling in a swine partial hindlimb model. Sci Rep.

[34] Berkane Y., Kostyra D.M., Chrelias T. (2023). The autonomization principle in vascularized flaps: an alternative strategy for composite tissue scaffold in vivo revascularization. Bioeng Basel Switz.

[35] Goutard M., Tawa P., Berkane Y. (2024). Machine perfusion enables 24-h preservation of vascularized composite allografts in a swine model of allotransplantation. Transpl Int Off J Eur Soc Organ Transplant.

[36] Liang H., Zhang P., Yu B. (2022). Machine perfusion combined with antibiotics prevents donor-derived infections caused by multidrug-resistant bacteria. Am J Transplant Off J Am Soc Transplant Am Soc Transpl Surg.

[37] Brouwers K., Kruit A.S., van Midden D. (2022). Ex vivo machine thrombolysis reduces rethrombosis rates in salvaged thrombosed myocutaneous flaps in swine. Plast Reconstr Surg.

[38] Oubari H., Van Dieren L., Berkane Y. (2025). 30. ex vivo preservation and study of a non-human primate partial face transplant model using sub normothermic machine perfusion. Transplantation.

[39] Dehnadi A., Rosales I.A., Xiong J.P. (2025). Inactivating the innate immune receptor CD11b with a first-in-class monoclonal antibody prolongs the survival of kidney allografts in nonhuman primates. Transplantation.

[40] Dehnadi A., Benedict Cosimi A., Neal Smith R. (2017). Prophylactic orthosteric inhibition of leukocyte integrin CD11b/CD18 prevents long-term fibrotic kidney failure in cynomolgus monkeys. Nat Commun.

[41] Yang Z.C., Lu W.Y., Geng Z.Y. (2025). Identification and validation of biomarkers related to mitochondria during ex vivo lung perfusion for lung transplants based on machine learning algorithm. Gene.

[42] Nakata K., Alderete I.S., Hughes B.A., Hartwig M.G. (2025). Ex vivo lung perfusion: recent advancements and future directions. Front Immunol.

[43] Brouwers K., Thijssen M.F., Kruit A.S. (2022). 24-hour perfusion of porcine myocutaneous flaps mitigates reperfusion injury: a 7-day follow-up study. Plast Reconstr Surg Glob Open.

[44] Haug V., Kollar B., Endo Y. (2020). Comparison of acellular solutions for ex-situ perfusion of amputated limbs. Mil Med.

[45] Krezdorn N., Macleod F., Tasigiorgos S. (2019). Twenty-four-hour ex vivo perfusion with acellular solution enables successful replantation of porcine forelimbs. Plast Reconstr Surg.

[46] Zhang L., Ipaktchi R., Ben Brahim B. (2024). Prolongation of the time window from traumatic limb amputation to replantation from 6 to 33 hours using ex vivo limb perfusion. Mil Med.

[47] Haug V., Kollar B., Tasigiorgos S. (2020). Hypothermic ex situ perfusion of human limbs with acellular solution for 24 hours. Transplantation.

[48] Werner N.L., Alghanem F., Rakestraw S.L. (2017). Ex situ perfusion of human limb allografts for 24 hours. Transplantation.

[49] Charlès L., Filz von Reiterdank I., Lancia H.H. (2024). Effect of subnormothermic machine perfusion on the preservation of vascularized composite allografts after prolonged warm ischemia. Transplantation.

[50] Filz Von Reiterdank I., Bento R., Dinicu A.T. (2026). Genetically engineered organs for early reporting of transplant rejection. Mol Ther.

[51] Filz Von Reiterdank I., Mojoudi M., Bento R. (2025). Ex vivo machine perfusion as a platform for lentiviral gene delivery in rat livers. Gene Ther.

[52] Oubari H., Lellouch A.G., Mojallal A., Cetrulo C.L., Uygun K., Berkane Y. (2025). Ex vivo preservation in vascularized composite allotransplantation: state of the art, challenges, and perspectives. Artif Organs.

[53] Dehnadi A., Benedict Cosimi A., Neal Smith R. (2017). Prophylactic orthosteric inhibition of leukocyte integrin CD11b/CD18 prevents long-term fibrotic kidney failure in cynomolgus monkeys. Nat Commun.

[54] Dehnadi A., Rosales I.A., Xiong J-P (2025). Inactivating the innate immune receptor CD11b with a first-in-class monoclonal antibody prolongs the survival of kidney allografts in nonhuman primates. Transplantation.

[55] Mattiassich G., Rittenschober F., Dorninger L. (2017). Long-term outcome following upper extremity replantation after major traumatic amputation. BMC Musculoskelet Disord.

[56] Malherbe M., Cheval D., Lejacques B., Vaiss L., Kerfant N., Le Nen D. (2013). Macro-amputation du membre supérieur: que sont devenus les patients ? À propos de 22 cas. Chir Main.

[57] Laing T.A., Cassell O., O'Donovan D., Eadie P. (2012). Long term functional results from major limb replantations. J Plast Reconstr Aesthet Surg.

[58] Gulgonen A., Ozer K. (2012). Long-term results of major upper extremity replantations. J Hand Surg (Eur Vol).

